# The three-dimensional stable mandibular landmarks in patients between the ages of 12.5 and 17.1 years

**DOI:** 10.1186/s12903-020-01142-2

**Published:** 2020-05-27

**Authors:** Gui Chen, Mona Al Awadi, David William Chambers, Manuel O. Lagravère-Vich, Tianmin Xu, Heesoo Oh

**Affiliations:** 1 Department of Orthodontics, Peking University School and Hospital of Stomatology, National Engineering Laboratory for Digital and Material Technology of Stomatology, Beijing Key Laboratory of Digital Stomatology, Beijing, 100081 China; 2 Department of Orthodontics, Arthur A. Dugoni School of Dentistry, University of the Pacific, 155 5th Street, San Francisco, CA 94103 USA; 3 Associate Professor of Department of Dentistry, Faculty of Medicine and Dentistry, University of Alberta, Edmonton, Alberta Canada

**Keywords:** Cone-beam CT, Mandibular growth, Stable landmarks, Symphysis, Mandibular canal

## Abstract

**Background:**

With the aid of implants, Björk identified two-dimensional mandibular stable structures in cephalograms during facial growth. However, we do not know what the three-dimensional stable structures are with certainty. The purpose of this study was to identify the most stable mandibular landmarks in growing patients using three-dimensional images.

**Methods:**

The sample was comprised of two cone-beam computed tomography (CBCT) scans taken about 4.6 years apart in 20 growing patients between the ages of 12.5 (T1) and 17.1 years (T2). After head orientation, landmarks were located on the chin (Pog), internal symphysis (Points C, D and E), and mandibular canals, which included the mental foramina (MF and MFA) and mandibular foramina (MdF). The linear distance change between Point C and these landmarks was measured on each CBCT to test stability through time. The reliability of the suggested stable landmarks was also evaluated.

**Results:**

The total distance changes between Point C and points D, E, Pog, MF, and MFA were all less than 1.0 mm from T1 to T2. The reliability measures of these landmarks, which were measured by the Cronbach alpha, were above 0.94 in all three dimensions for each landmark. From T1 to T2, the distance changes from Point C to the right and left mandibular foramina were 3.39 ± 3.29 mm and 3.03 ± 2.83 mm, respectively.

**Conclusions:**

During a growth period that averaged 4.6 years, ranging from 11.2 to 19.8 years old, the structures that appeared relatively stable and could be used in mandibular regional superimpositions included Pog, landmarks on the inferior part of the internal symphysis, and the mental foramen. The centers of the mandibular foramina and the starting points of the mandibular canal underwent significant changes in the transverse and sagittal dimensions.

## Background

A challenge in measuring growth is identifying a stable reference point. Measuring change is complicated by not being able to determine whether one or both landmarks have shifted in position relative to the overall matrix of interest. There are no means to analyze the sources of change without having an external reference point.

Björk was the first person who studied facial growth using metallic implants as an external reference point [1–3]. He also identified natural stable structures of the maxilla and mandible in cephalograms. In the mandible, the tip of the chin and the following three internal structures are considered stable: (1) the inner cortical structure of the inferior border of the symphysis, (2) detailed structures from the mandibular canal, and (3) the lower contour of the molar germ from the time that mineralization of the crown is visible until the roots begin to form [2]. All of these stable regions are projections of three- dimensional (3D) structures onto two-dimensional (2D) lateral films and are difficult to accurately relate to the original anatomy, except structures located in the midsagittal plane.

Ruellas found that the Björk registration could not work properly in most 3D mandibular superimposition cases using CBCT images [4]. One of the reasons discussed was the displacement of the mandibular canal and other “stable structures” as a result of growth. Due to this inherent shortcoming of the 2D image, the transversal growth information of the mandibular canal is absent in the lateral x-ray film and minimally present in the posterior-anterior cephalogram. Fortunately, CBCT overcomes this limitation.

CBCT offers a valid 3D representation of the skeletal structures of the cranium and employs much less radiation than computed tomography (CT) scans [5]. With the aid of CBCT, the transversal change of facial growth can be evaluated to identify additional reference structures. However, assessing the change in growing patients is still a challenge since stable natural structures of the maxilla and mandible are difficult to identify without a unique implant CBCT sample similar to that of Björk’s.

Recently, Nguyen identified 3-dimensionally stable mandibular structures in growing patients with the aid of bone plates and found some anterior stable areas (the chin and symphysis regions) [6]. However, it has been questioned whether some stable structures posterior to the symphysis region can be identified to better perform mandibular superimpositions.

The objective of this study was to identify natural stable references in the mandible using longitudinal CBCT data. With the aid of these stable landmarks, the accuracy of different mandibular superimposition methods could be tested.

## Methods

This investigation was a retrospective observational longitudinal study.

**Fig. 1:**
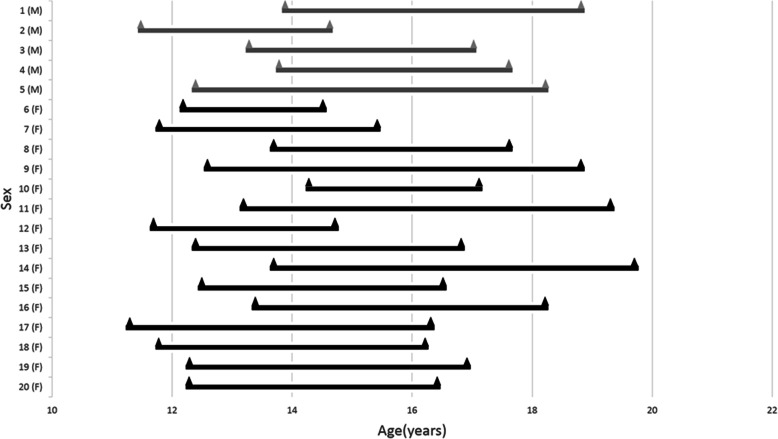
Sample distribution by sex and age. The length of the line indicates the time interval between two CBCTs

The study sample consisted of a total of 20 adolescent patients from a maxillary expansion randomized clinical trial conducted at the University of Alberta, Canada [7]. The inclusion criteria were a period of at least 2.5 years between two CBCTs and no mandibular jaw surgery. Cases in the sample were from both the expander and control groups, since we focused on the mandible. The exclusion criteria included poor image quality and mandibular asymmetry with greater than 2 mm of chin deviation. Severe asymmetrical patients were excluded because the symphysis of the mandible may not coincide with the midsagittal plane of the mandible due to chin deviation. The sample included 5 boys and 15 girls. The mean age at the initial CBCT (T1) was 12.5 ± 0.9 years (11.2 to 14.2 years) and the mean age at the final CBCT (T2) was 17.1 ± 1.5 years (14.6 to 19.8 years). The mean time interval between the two CBCTs was 4.7 ± 1.1 years, ranging from 2.5 to 6.6 years (Fig. 1).

CBCT scans at T1 were taken using a NewTom 3G (Aperio Services, Verona, Italy) at 110 kV, 6.19 mAs, 8 mm aluminum filtration, and voxel size of 0.25.

**Fig. 2:**
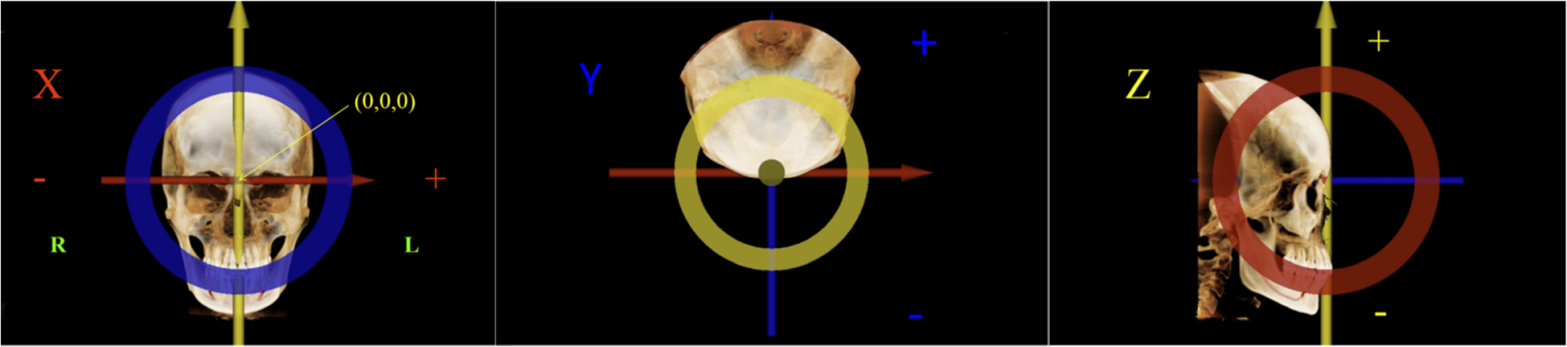
Head orientation before placing landmarks. Frankfort plane is constructed with the X-axis and Y-axis. Sagittal plane is constructed with the Y-axis and Z-axis. Frontal plane is constructed with the X-axis and Z-axis. X = Right (−)-Left (+).Y = Antero (−)-Posterior (+). Z = Superior (+)-Inferior (−).The origin is at point N

Mm, while scans at T2 were taken using the iCAT machine (Imaging Sciences International, Hatfield, Pa) with a collimation height scan of 13 cm, 120 kV, 24 mA, scan time of 20 s, and voxel size of 0.3 mm. The DICOM files were imported into the Invivo 6.0 software program (Anatomage, San Jose, CA) to display the images. The orientation of the volumetric images was performed by using three reference planes: (1) Frankfort Horizontal plane (FH) – the primary reference plane that intersects right Porion (Po), left Po, and right Orbitale (Or); (2) Midsagittal plane – plane passing through Nasion (N) and Basion (Ba) that is perpendicular to FH; and (3) Frontal plane – plane perpendicular to both the FH and midsagittal planes and passing through N. N point was set as the origin (Fig. 2 and Table 1).

**Table 1:** Landmark names and definitions

Landmark	Symbol	Definition	Category
Nasion	N	The most antero-inferior point on the frontal bone at the fronto-nasal suture.	Landmarks for head orientation
Orbitale	Or	The most inferior point of the lower border of the boney orbit.	
Porion	Po	The most superior point of external auditory meatus.	
Basion	Ba	The most inferior point at the anterior margin of the foramen magnum in the midsagittal plane.	
Pogonion	Pog	The most anterior point in mandibular chin area in the sagittal plane.	Landmarks on chin
Point C	C	The deepest point on the anterior surface of the lingual cortical plate of the chin symphysis (Fig. 3).	Reference landmarks in symphysis
Point D	D	Intersection of the line connecting C and Pog with the anterior cortical plate of the chin (Fig. 3).	
Point E	E	The most inferior point on the inner surface of the cortical plate of the chin symphysis (Fig. 3).	
Mental foramen	MF	The center of the mental foramen (Fig. 4).	Landmarks on mandibular canal
Anterior mental foramen	MFA	The most anterior point of the mental foramen (Fig. 4).	
Mandibular foramen	MdF	The center of the mandibular foramen (Fig. 4).	

**Fig. 3:**
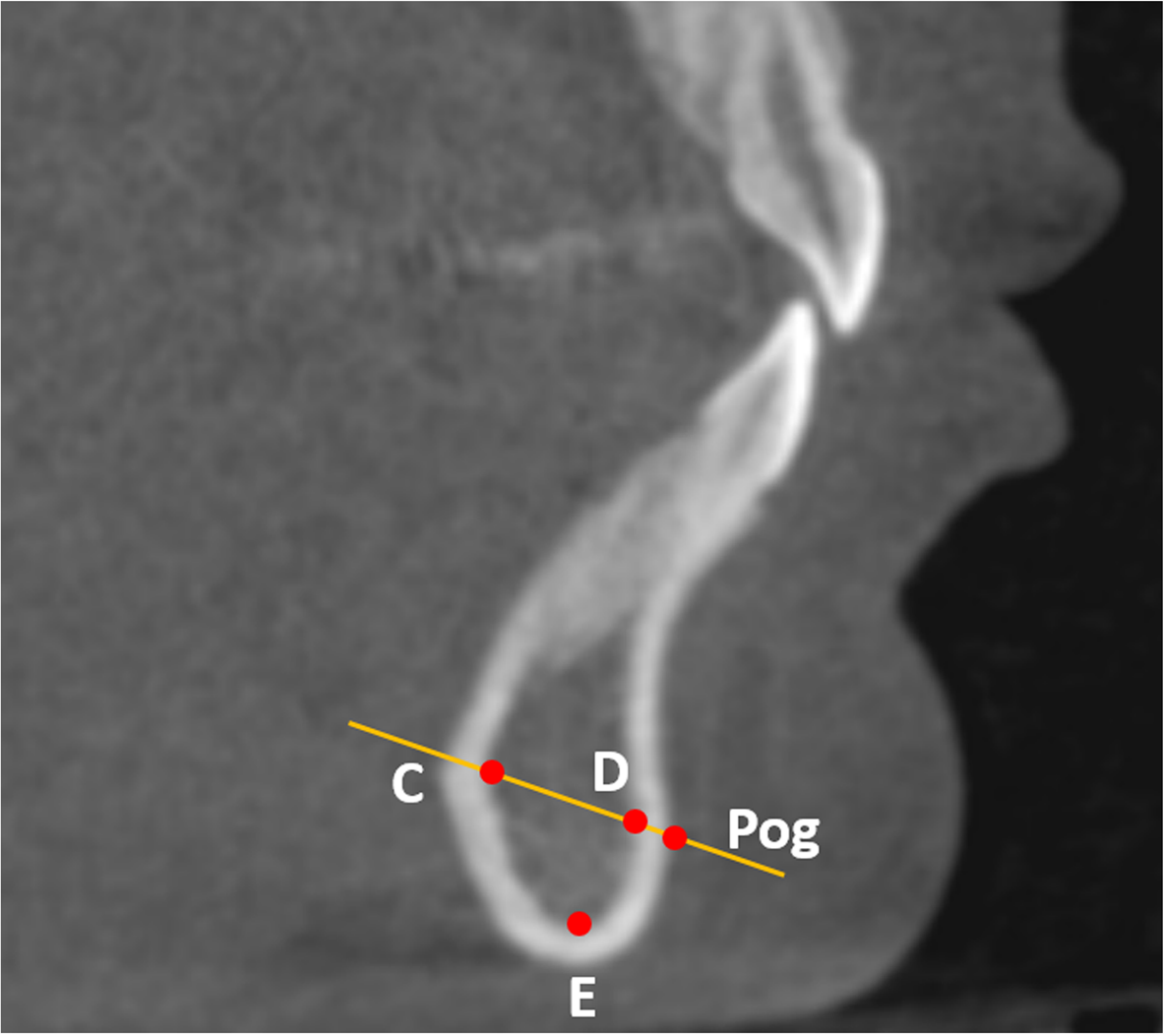
The positions of C, D, E, and Pog and their relationship. C, D,and E are located in the middle sagittal plane of the mandible, which is parallel to the midsagittal plane of the head and passes through Pog

**Fig. 4:**
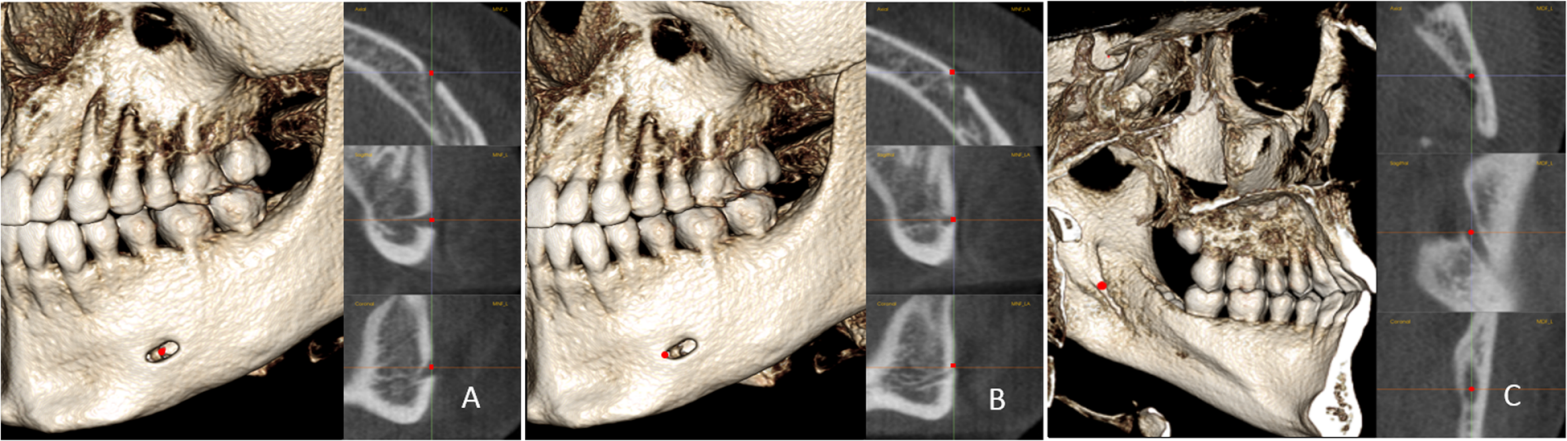
The positions of MF, MFA, and MdF on the left side: **a**, MF is located in center of the mental foramen; **b**, MFA is the most anterior point of the mental foramen; **c**, MdF is located in the center of the mandibular foramen. It is placed on the first slice where the canal shows a complete circle from superior to inferior

Following Björk and Skieller’s method in their study on stable structures of the mandible using 2D lateral cephalograms [3], 10 skeletal landmarks were selected: Pog; Points C, D, and E on the internal symphysis; and MF, MFA, and MdF on the mandibular canal (Table 1 and Figs. 3, 4). The positions of C, D, and E were located on the midsagittal plane of the mandible. Landmark locations were checked in both volumetric and sectional views. The calibration process for landmark location was performed by two judges using randomly selected cases. After satisfactory calibration sessions, each image was traced by 2 calibrated judges and the estimates were averaged when reporting all measurements.

**Fig. 5:**
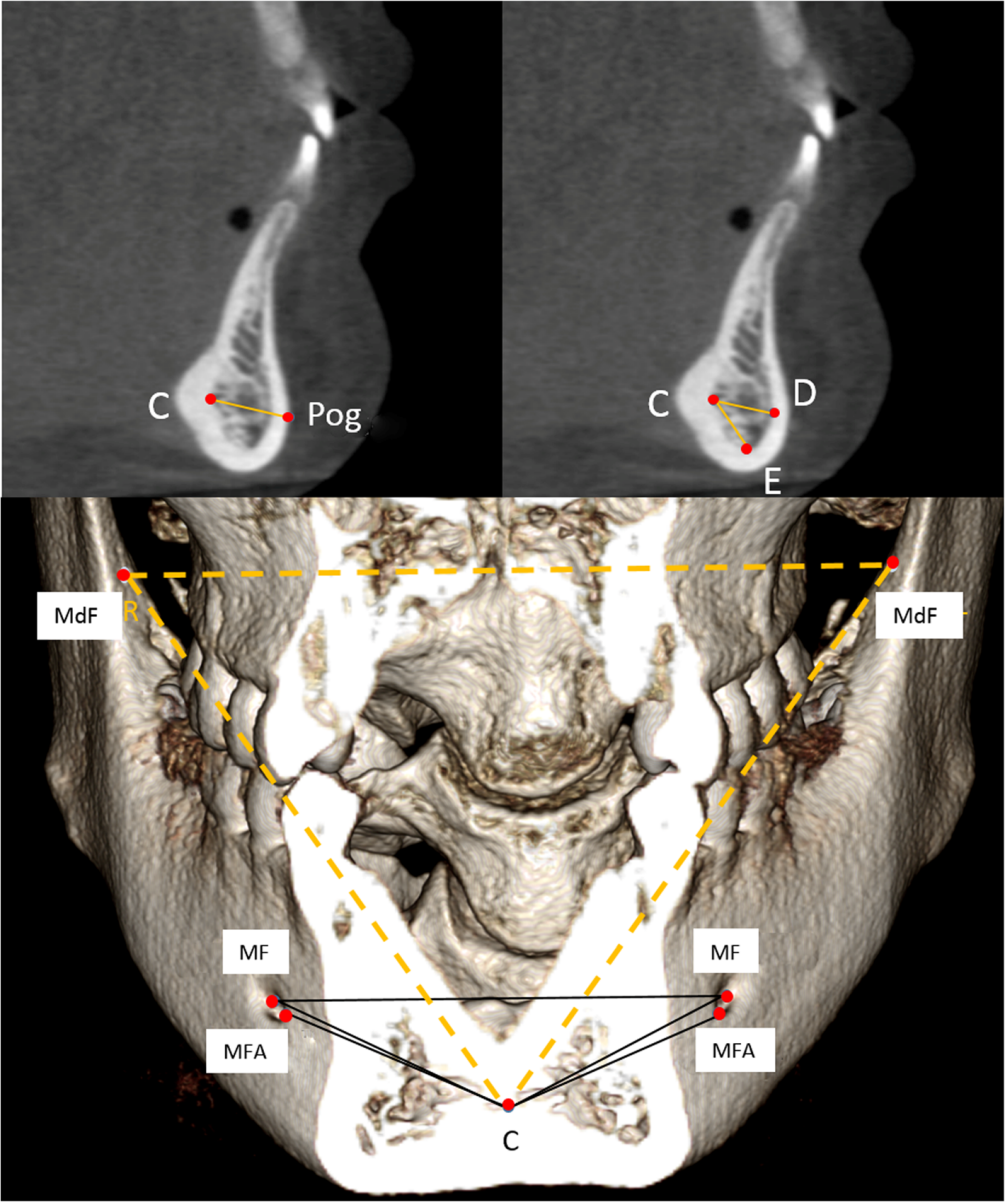
Eleven linear measurements in this study

Landmarks C, D, and E, located in the inner cortical structure of the symphysis, were selected to represent the natural reference points in the symphysis. C indicates the most posterior point of the internal symphysis [4], D indicates the most anterior point of pogonion on the internal symphysis, and E indicates the most inferior point of the internal symphysis. The Invivo 3D custom analysis tool was used to calculate the distance between the landmarks and reference points C, D, E in 3D space using the formula d = square root of [(x_1_ -x_2_)^2^ + (y_1_-y_2_)^2^ + (z_1_-z_2_)^2^] (Fig. 5). X, y, and z are the coordinates of the landmarks.

### Statistical analysis

In addition to descriptive statistics used to characterize the initial and final locations, changes between the reference points and 7 landmarks, t-tests for differences in mean landmark change, and linear and polynomic regression analyses were used to project the location and stability of the theoretical parameters of C, D, and E. The consistency of two judges was determined by Cronbach’s alpha, regression analysis, and a normalized score for differences between judges [8]. Sources of variance (patient, time, judge, and error) were estimated using Cronbach’s generalizability method [9]. SPSS 22.0 for Windows (IBM Corp, Armonk, NY) was used for all statistical analyses.

## Results

### Consistency of the judges

The inter-judge reliability of the landmarks, which was tested using the Cronbach α, was above 0.94 in all three dimensions for all landmarks (Table 2). Even when consistency between judges was high, bias may remain [8]. If one gives proportionally higher scores to extreme values, the intraclass correlation coefficient (ICC) will remain high, but the regression lines between judges will not be parallel. If one judge consistently sees each distance as greater than the other judge does, the average scores will show a displacement even though the ICC may be excellent. The Cronbach α (equivalent to ICC in this case) and the slope of the regression line for judge scores should both be 1.0, and the displacement should be 0.0.

**Table 2:** Inter-examiner reliability of the landmarks

Landmark		X			Y			Z		
		α	Slope	Displacement (mm)	α	Slope	Displacement (mm)	α	Slope	Displacement (mm)
Pog		0.955	0.971	− 0.158	0.997	0.976	−0.010	0.998	0.988	0.018
C		0.952	0.965	−0.170	0.996	0.981	−0.010	0.997	0.996	0.020
D		0.962	0.947	−0.160	0.998	0.995	0.019	0.988	0.980	0.000
E		0.947	0.921	−0.064	0.997	0.980	0.008	0.999	1.000	0.000
Right	MF	0.976	0.932	−0.049	0.995	0.987	0.013	0.997	1.018	0.028
	MFA	0.983	0.951	−0.037	0.996	0.992	0.009	0.998	0.999	0.047
	MdF	0.983	0.938	−0.016	0.991	0.974	0.061	0.993	1.006	0.037
Left	MF	0.983	0.972	0.019	0.997	0.981	0.010	0.997	0.995	0.034
	MFA	0.983	0.938	−0.025	0.997	0.985	0.007	0.998	1.008	0.021
	MdF	0.964	0.995	−0.013	0.992	0.984	0.009	0.994	0.971	0.010

### Stability test among C, D, and E

**Fig. 6:**
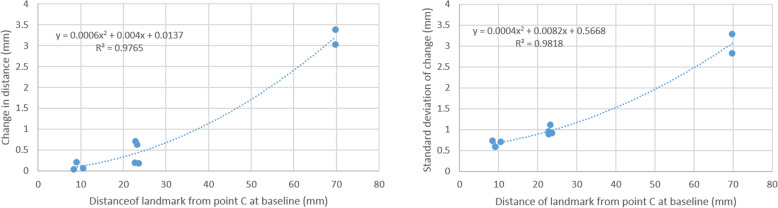
Relationship between distance from reference point C and 9 landmarks and change in landmark from T1 to T2 (on left) and standards deviation of that change (on right)

The approach used to establish the stability of C, D, and E was to project an ideal reference point, one that minimizes variation, from nine other landmarks on the mandible. In order to be considered stable, the measured landmark should be very close to the projection from other landmarks of interest. Two projections were made: one to find the best reference point based on change in other landmarks and the other based on the standard deviation of those changes (Fig. 6). Three parameters can be used to characterize the quality of the projections. R-values reflect the adequacy of the projections. The intercept of the regression line (the point at which the distance between the target landmark and the reference is 0.0) is the 3D distance between the actual reference point and the theoretically projected one. The average 3D distance between the actual and projected reference point can be calculated algebraically from the distance equation.

The estimates for differences between ideal projections and actual reference points are shown in Table 3. Generally, C is most stable with an R-value of 0.988, intercept of 0.014, and average deviation of 0.008 mm (Table 3).

**Table 3:** The estimates for difference between ideal projections and actual reference points (Point C, D, and E)

	C			D			E		
	R	Intercept (mm)	Ave Dev (mm)	R	Intercept (mm)	Ave Dev (mm)	R	Intercept (mm)	Ave Dev (mm)
Change	0.988	0.014	0.008	0.976	0.095	0.055	0.988	0.097	0.056
SD	0.991	0.569	0.327	0.991	0.652	0.376	0.972	0.246	0.143

R values reflect the adequacy of the projections

### Changes in landmarks between T1 and T2

Table 4 shows 3D linear distances between 9 landmarks and C, as well as the changes in these values from T1 to T2. Landmarks that were more distant from C at T1 exhibited both the greatest change and the largest standard deviations in change. Interestingly, the mental foramen position seems relatively stable, with less than 1.0 mm of change in both its distance to the reference point in the symphysis and the width between the right and left mental foramina. On the other hand, the mandibular foramen underwent significant changes in both the transverse and sagittal dimensions.

**Table 4:** Distance change among the landmarks (mm) between T1 and T2 (*N* = 20)

Measurements		T1		T2		T1-T2		*T*	*P*
		Mean	SD	Mean	SD	Mean	SD		
Age (Year)		12.6	0.9	17.1	1.5	4.5	1.1	17.857	<.001
C-D		8.37	1.49	8.40	1.46	0.03	0.73	0.176	NS
C-E		9.04	1.85	9.25	1.84	0.21	0.59	1.631	NS
C-Pog		10.53	1.50	10.59	1.48	0.06	0.71	0.368	NS
Right	C-MF	23.63	1.79	23.81	1.93	0.18	0.92	0.853	NS
	C-MFA	22.57	1.56	22.77	1.81	0.20	0.89	0.980	NS
	C-MdF	69.73	3.66	72.76	4.90	3.03	2.83	4.669	<.001
Left	C-MF	23.21	1.92	23.84	1.86	0.63	1.11	2.471	0.050
	C-MFA	22.14	1.86	22.85	1.64	0.71	0.95	3.257	0.010
	C-MdF	69.77	3.78	73.16	4.46	3.39	3.29	4.490	<.001
MF -MF		43.58	2.54	44.02	2.40	0.44	0.77	2.490	0.050
MdF -MdF		78.74	3.23	81.30	3.78	2.56	2.08	5.367	<.001

NS at *P* ≥ 0.05

### Partitioning sources of variance in judged distance measurement

Table 5 and Fig. 6 display results reflecting the consistency of measurements involving change. Table 5 is based on a 3-way ANOVA with patients, judge, and time as factors. The significance columns can be interpreted in the usual fashion. For example, averages that combine T1 and T2 are significant across subjects for all distances, meaning that some mandibles are larger than others. The patient x time interaction is also significant for all landmarks, showing that the rate of growth differs across subjects in all measured dimensions.

**Table 5:** Three way ANOVA analysis of the distance change among the landmarks between T1 and T2

	C-D		C-E		C-Pog		Right						Left						MF-MF		MdF -MdF	
							C-MF		C-MFA		C-MdF		C-MF		C-MFA		C-MdF					
	Sig	% var	Sig	% var	Sig	% var	Sig	% var	Sig	% var	Sig	% var	Sig	% var	Sig	% var	Sig	% var	Sig	% var	Sig	% var
Patient (P)	0.000	84	0.000	88	0.000	86	0.000	79	0.000	81	0.000	64	0.000	75	0.000	76	0.000	52	0.000	91	0.000	65
Time (T)	_	0	0.100	0	_	0	_	0	_	0	0.000	19	0.020	4	0.003	7	0.000	24	_	1	0.000	20
Judge (J)	_	0	_	0	_	0	_	1	0.070	1	_	_	_	1	_	1	_	_	_	0	_	_
P x T	0.005	8	0.003	3	0.000	9	0.020	7	0.000	11	0.000	16	0.005	11	0.004	10	0.000	23	0.040	3	0.000	13
P x J	_	0	0.007	5	_	1	0.060	5	0.040	3	_	_	_	0	_	1	0.080	_	_	1	0.080	1
T x J	_	0	_	0	_	0	_	0	_	0	_	_	_	0	_	0	_	_	_	0	_	
e	_	7	_	4	_	4	_	8	_	5	_	2	_	9	_	7	_	1	_	4	_	1
Cronbach α		0.934		0.947		0.935		0.908		0.908		0.884		0.906		0.916		0.812		0.970		0.904
CI_95_		0.728		0.820		0.723		1.095		0.985		4.007		1.253		1.175		4.597		0.917		3.219

Sig: *P*-value from three-way ANOVA, only values significant less than or equal to 0.10 shown

% var.: Proportion of variance attributalbe to each compoent

e:error, the name for all unmeasured sources

α: ICC

CI_95_: Approximately 95% of true means with fall in the range ± CI_95_

Cronbach’s generalizability method allows for the partitioning of variance by source. This is displayed in Table 5 under the column headings for “% var.” Consistently, the largest sources of variance in growth are patient differences, followed by patient-by-time interaction and error. Time was not an important factor in contributing to the distance change from C to D, E, Pog, and mental foramen. However, time was an important factor for the distance change from C to the mandibular foramen on each side. The mean distance changes were about 3.0 mm over 4.6 years.

**Fig. 7:**
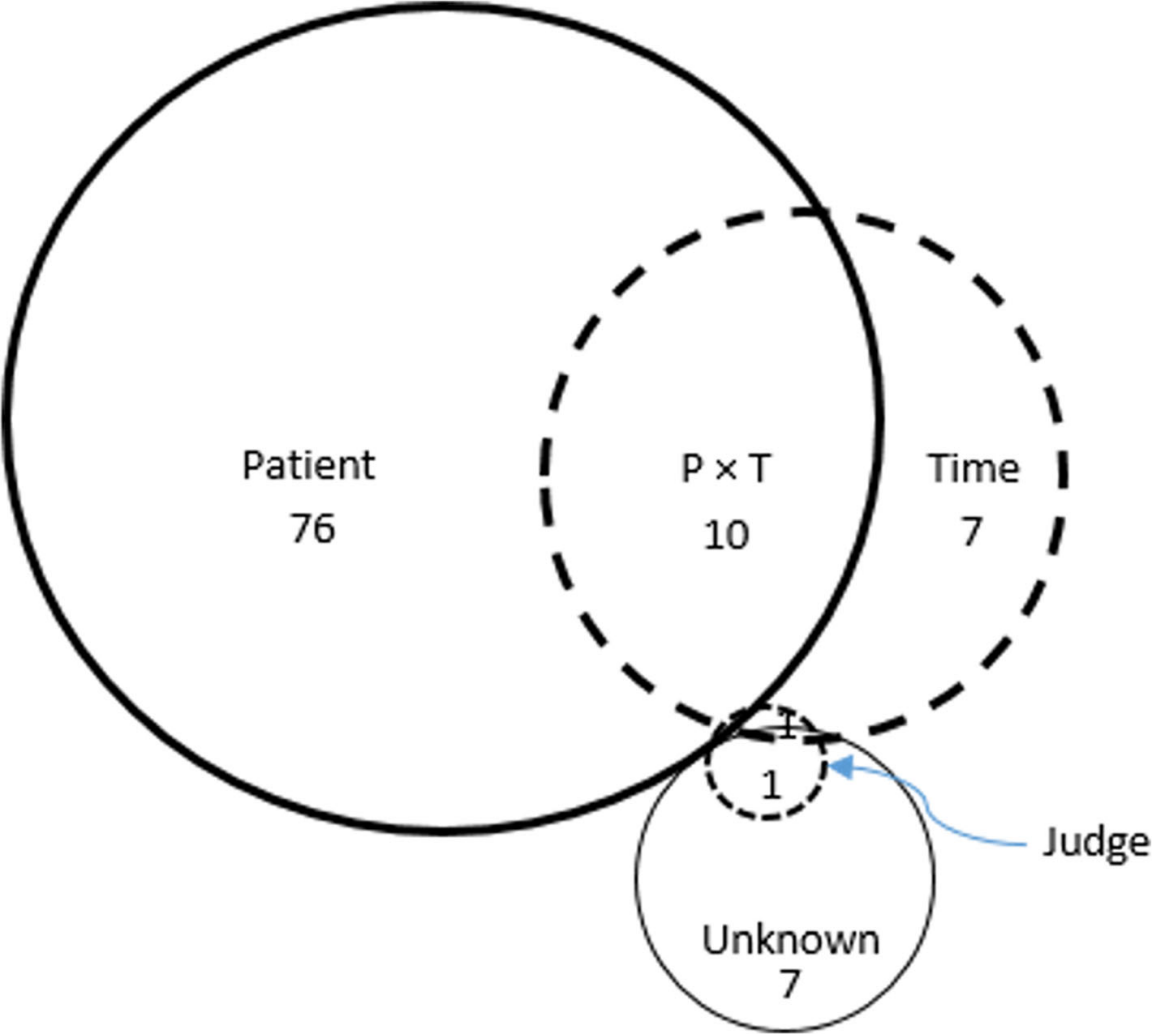
Variance analysis: Proportion of variance for each component of the C-MFA (left). Numbers represent percentages. Patients contributed 76%, time contributed 7%, and judge contributed 1%; patient x time contributed 10%, patient x judge contributed 1%, and judge x time contributed 0%. The unknown factors (e) contributed the last 7% of variance

The partitioning of sources of variance for the example of C-MFA (left) is shown graphically in Fig. 7, where the area of each segment is meant to be proportional to the proportion of variance associated with that source or combination of sources.

## Discussion

CBCT has been proven to be a valid 3D representation of the skull that is suitable for clinical and laboratorial use. To evaluate mandibular change properly, CBCT superimposition is supposed to be the best method. The voxel-based method was first described in dentistry by Cevidanes et al. [10] and became the most popular. Voxel-based superimposition matches the grayscale values of the voxels to superimpose the CBCT images. Ruellas et al. [4] suggested superimposing the mandible in growing patients on the mandibular body (mandible without teeth, alveolar bone, rami and condyles) using 3D regional registration. Koerich et al. [11] showed that this was a precise method for 3D mandibular superimposition in growing subjects.

However, there may be concerns about using the entire mandibular body to superimpose growing patients because certain areas of the mandibular body are remodeled during growth, such as the chin and the lingual tuberosity, which may not qualify as reference structures [12]. Erroneous information regarding patterns of bone growth and remodeling would be obtained if biologically incorrect superimposition protocols are used [13]. Ideally, superimposition should be based on the most stable, verified structure with good reliability.

In past studies using 2D cephalograms, stable structures in the mandible were identified with the aid of metallic implants [1–3], which can unlikely be repeated nowadays using CBCT. Fortunately, the inner cortical structure of the inferior border of the mandibular symphysis, located on the facial midline and having no transversal change during growth, was verified by implants to be stable sagittally and vertically. In this study, we used this structure as an indirect reference to identify other stable landmarks or structures in the mandible.

After several trials and errors, we found eight relatively stable landmarks. Among them, points C, D, E and Pog were located on the stable regions, which was in agreement with Nguyen’s study. With the aid of bone plates and screws, Nguyen et al. found that the chin and symphysis regions were stable areas for 3-dimensional mandibular regional superimpositions [6]. In addition, two new landmarks (MF and MFA) on the mental foramina on each side, which cannot be viewed in lateral cephalograms, were identified. In the present study, the positional stability of C, D, and E through time was statistically tested. All three points showed high stability over a 4.6-year time interval. In addition, C turned out to be the most stable point and was used as the reference point to measure the stability of other landmarks in the mandible. The average changes in distance from C to all of the reference landmarks were less than 1.0 mm, and the change in width between the right and left mental foramina (MF- MF) was 0.44 mm, which is a relatively small change during 4.6 years of growth.

In this study, a three-way ANOVA, which gave more detailed insight into how this difference may be occurring (Table 5, Fig. 6), was used to analyze the distance change between T1 and T2. The statistical analysis of the change in C-D, C-E, C-Pog, C-MF, C-MFA, and MF-MF showed that time was not an important factor in contributing to the distance change, indicating the stability of these landmarks during growth.

Reliability is another important characteristic of the reference landmark. The Cronbach α values for the 8 relatively stable landmarks were all above 0.94 in all 3 dimensions and did not exhibit scale distortion or discrepancy bias.

In this study, we estimated the dimensional change of the mandibular canal by quantifying the positional stability of the mandibular foramen (starting point of the canal) and mental foramen (terminal point of the canal). The position of the mental foramina appears stable in all 3 dimensions, with less than 1.0 mm of change. However, the position of the mandibular foramina shows some changes, such as an increase of 2.5 ± 2.1 mm transversally and an increase of about 3.0 mm linearly from C to the right and left mandibular foramina. This finding is in agreement with the result of Krarup’s study, which analyzed normal mandibular growth using medical CTs in 10 children with Apert syndrome from 1 week to 14.5 years [14]. The mandibular canals were relocated laterally; therefore, we should be careful in including the posterior part of the mandibular canal as a reference structure for mandibular regional superimposition. This information can provide an important foundation for mandibular regional superimposition using both methods based on landmarks or voxels.

No landmark on the molar germs was mentioned in this study, since in most cases the roots of the third molars started developing at T2. Furthermore, it is difficult to place a reliable landmark on the lower contour of the molar germ, which is a smooth and curved surface in the early stages.

### Limitations

The present study has some limitations. There were more girls than boys, and we did not include younger age groups in the sample. These limitations were due to the availability of data. Although mandibular superimposition using four symphysis landmarks and four landmarks at the mental foramina on both the right and left sides should theoretically suffice for the superimposition of 3D images, the landmark-based superimposition method is less accurate than the voxel-based method [15]. The reason is that landmark identification on 3D images is complex. The present study could not answer whether the mandibular canal gradually displaces laterally, or that a significant displacement occurs in a specific part of the mandibular canal. In a future study, we plan to explore more stable structures based on the stable landmarks we have identified to improve the voxel-based superimposition method in growing patients and to investigate 3D growth of the mandibular canals. In addition, further validation studies against other samples are required for the suggested stable landmarks in the present study.

## Conclusions

We assessed the stability of mandibular structures in the symphysis and mandibular canal during an average growth period of 4.6 years, ranging from 11.2 to 19.8 years, We also introduced new stable landmarks for mandibular superimposition. Pog, landmarks on the inferior part of the internal symphysis (C, D, and E), and the mental foramen appear relatively stable, and thus, can be used in mandibular regional superimpositions. The mandibular foramina showed significant positional changes in both the transverse and sagittal dimensions.

## Data Availability

The datasets used and analyzed during the current study are available from the corresponding authors upon reasonable request.

## Abbreviations

3D: Three-dimensional
2D: Two-dimensional
Ba: Basion
CT: Computed tomography
FH: Frankfort Horizontal plane
ICC: Intraclass correlation coefficient
N: Nasion
Or: Orbitale
Po: Porion
